# Updated checklist of Azores Actinopterygii (Gnathostomata: Osteichthyes)

**DOI:** 10.3897/BDJ.9.e62812

**Published:** 2021-03-11

**Authors:** Luís M. D. Barcelos, José M. N. Azevedo, João Pedro Barreiros

**Affiliations:** 1 Universidade dos Açores, Angra do Heroísmo, Portugal Universidade dos Açores Angra do Heroísmo Portugal; 2 ce3c - Centre for Ecology, Evolution and Environmental, Angra do Heroísmo, Portugal ce3c - Centre for Ecology, Evolution and Environmental Angra do Heroísmo Portugal; 3 University of the Azores, Ponta Delgada, Portugal University of the Azores Ponta Delgada Portugal; 4 ce3c - Centre for Ecology, Evolution and Environmental, Ponta Delgada, Portugal ce3c - Centre for Ecology, Evolution and Environmental Ponta Delgada Portugal

**Keywords:** checklist, fin fishes, new records, regional inventory, taxonomic update

## Abstract

**Background:**

Since the first published comprehensive checklist of Azorean fishes - covering the whole Exclusive Economic Zone (EEZ) region - several new records have been published and an updated checklist published in 2010. This new dataset covers all confirmed species of actinopterygians for the Azorean EEZ.

**New information:**

In this update, we made corrections to the previous checklists, updated the taxonomy according to the most recent bibliography and added two new species to the Azorean Actinopterygii checklist.

## Introduction

There are over 16,000 fish species distributed by 85 orders and 536 families (Nelson et al. (2016). The Actinopterygii, commonly known as ray-finned fishes, are the largest group of living fishes. Although this group is well studied, it is still possible to discover new species or report new records for some areas.

Based on the previous list (Porteiro et al. 2010), we provide here an updated taxonomic classification following Fricke et al. (2021) and species records, by adding new records (Barreiros et al. 2014, Ribeiro et al. 2017, Barros-García et al. 2018), resulting in this checklist, already online on GBIF (Barcelos et al. 2020). This information can also be found in Suppl. material 1.

## General description

### Purpose

Updating Azorean biota checklists.

## Project description

### Title

Azores Actinopterygii - updated checklist

### Personnel

Luís Barcelos, José Azevedo, João Barreiros

### Study area description

Azores Archipelago

### Funding

Funding Institutions: Azores PO 2020 - ACORES-01-0145-FEDER-000072; TOTAL BUDGET: €299,901.83, EU Support: €254,916.56. This project was financed by FEDER in 85% and by Azorean Public funds by 15% through the Operational Programme Azores 2020. This work is also funded by FEDER funds through the COMPETE 2020 Programme and National Funds through FCT - Portuguese Foundation for Science and Technology under the Research Infrastructure PORBIOTA - Portuguese E-Infrastructure for Information and Research on Biodiversity, project number POCI-01-0145-FEDER-022127.

## Geographic coverage

### Description

Azores EEZ (Economic Exclusive Zone) limits.

### Coordinates

34 and 42.976 Latitude; -35.578 and -21 Longitude.

## Taxonomic coverage

### Description

The dataset covers all the ray-finned fishes so far known to occur within the Azores' EEZ. Here, we present a list to the family taxonomic level. For each species and other information, see the Supplementary file.

### Taxa included

**Table:** 

Rank	Scientific Name	Common Name
kingdom	Animalia	Animals
phylum	Chordata	
class	Actinopterygii	Ray-finned fishes
order	Tetraodontiformes	Puffers and filefishes
order	Pleuronectiformes	Flatfishes
order	Gobiesociformes	Clingfishes
order	Perciformes	Perch-likes
order	Scorpaeniformes	Scorpionfishes and flatheads
order	Syngnathiformes	Pipefishes and seahorses
order	Zeiformes	Dories
order	Beryciformes	Sawbellies
order	Cetomimiformes	Whalefishes
order	Stephanoberyciformes	ricklefishes, bigscales and gibberfishes
order	Beloniformes	Needle fishes
order	Atheriniformes	Silversides
order	Mugiliformes	Mullets
order	Lophiiformes	Anglerfishes
order	Ophidiiformes	Cusk eels
order	Gadiformes	Cods
order	Polymixiiformes	Beardfishes
order	Lampridiformes	Velifers, tube-eyes and ribbonfishes
order	Myctophiformes	Lanternfishes
order	Aulopiformes	Grinners
order	Stomiiformes	Lightfishes and dragonfishes
order	Osmeriformes	Smelts
order	Clupeiformes	Herrings
order	Saccopharyngiformes	Swallowers and gulpers
order	Anguilliformes	Eels and morays
order	Notacanthiformes	Halosaurs and deep-sea spiny eels
order	Elopiformes	Tarpons and tenpounders
family	Molidae	
family	Diodontidae	
family	Tetraodontidae	
family	Ostraciidae	
family	Monacanthidae	
family	Balistidae	
family	Cynoglossidae	
family	Bothidae	
family	Scophthalmidae	
family	Gobiesocidae	
family	Caproidae	
family	Tetragonuridae	
family	Nomeidae	
family	Centrolophidae	
family	Istiophoridae	
family	Xiphiidae	
family	Scombridae	
family	Trichiuridae	
family	Gempylidae	
family	Sphyraenidae	
family	Scombrolabracidae	
family	Luvaridae	
family	Gobiidae	
family	Callionymidae	
family	Blenniidae	
family	Tripterygiidae	
family	Trachinidae	
family	Ammodytidae	
family	Chiasmodontidae	
family	Zoarcidae	
family	Scaridae	
family	Labridae	
family	Pomacentridae	
family	Chaetodontidae	
family	Kyphosidae	
family	Mullidae	
family	Sparidae	
family	Haemulidae	
family	Lobotidae	
family	Caristiidae	
family	Lutjanidae	
family	Bramidae	
family	Carangidae	
family	Echeneidae	
family	Coryphaenidae	
family	Pomatomidae	
family	Epigonidae	
family	Apogonidae	
family	Priacanthidae	
family	Callanthiidae	
family	Serranidae	
family	Epinephelidae	
family	Polyprionidae	
family	Howellidae	
family	Triglidae	
family	Sebastidae	
family	Setarchidae	
family	Scorpaenidae	
family	Dactylopteridae	
family	Centriscidae	
family	Fistulariidae	
family	Syngnathidae	
family	Zeidae	
family	Grammicolepididae	
family	Parazenidae	
family	Oreosomatidae	
family	Berycidae	
family	Trachichthyidae	
family	Diretmidae	
family	Anoplogastridae	
family	Rondeletiidae	
family	Stephanoberycidae	
family	Melamphaidae	
family	Scomberesocidae	
family	Belonidae	
family	Exocoetidae	
family	Atherinidae	
family	Mugilidae	
family	Linophrynidae	
family	Ceratiidae	
family	Oneirodidae	
family	Himantolophidae	
family	Melanocetidae	
family	Caulophrynidae	
family	Chaunacidae	
family	Antennariidae	
family	Lophiidae	
family	Bythitidae	
family	Ophidiidae	
family	Carapidae	
family	Lotidae	
family	Gadidae	
family	Phycidae	
family	Melanonidae	
family	Moridae	
family	Macrouridae	
family	Polymixiidae	
family	Regalecidae	
family	Stylephoridae	
family	Trachipteridae	
family	Radiicephalidae	
family	Lampridae	
family	Myctophidae	
family	Neoscopelidae	
family	Bathysauridae	
family	Paralepididae	
family	Anotopteridae	
family	Omosudidae	
family	Alepisauridae	
family	Evermannellidae	
family	Scopelarchidae	
family	Ipnopidae	
family	Notosudidae	
family	Chlorophthalmidae	
family	Synodontidae	
family	Aulopidae	
family	Stomiidae	
family	Phosichthyidae	
family	Sternoptychidae	
family	Gonostomatidae	
family	Alepocephalidae	
family	Platytroctidae	
family	Microstomatidae	
family	Bathylagidae	
family	Opisthoproctidae	
family	Clupeidae	
family	Eurypharyngidae	
family	Saccopharyngidae	
family	Cyematidae	
family	Serrivomeridae	
family	Nettastomatidae	
family	Congridae	
family	Nemichthyidae	
family	Derichthyidae	
family	Ophichthidae	
family	Synaphobranchidae	
family	Muraenidae	
family	Chlopsidae	
family	Anguillidae	
family	Notacanthidae	
family	Halosauridae	
family	Megalopidae	

## Traits coverage

### Data coverage of traits

PLEASE FILL IN TRAIT INFORMATION HERE

## Usage licence

### Usage licence

Creative Commons Public Domain Waiver (CC-Zero)

### IP rights notes

This work is licensed under a Creative Commons Attribution (CC-BY) 4.0 Licence.

## Data resources

### Data package title

Azores Actinopterygii updated checklist

### Resource link

http://ipt.gbif.pt/ipt/resource?r=actinopterygii_azores_checklist_vs1_2019&v=1.5

### Alternative identifiers

http://ipt.gbif.pt/ipt/resource?r=actinopterygii_azores_checklist_vs1_2019

### Number of data sets

1

### Data set 1.

#### Data set name

Azores Actinopterygii updated checklist

#### Data format

Darwin Core

#### Number of columns

14

#### Description

Since the first published comprehensive checklist of Azorean fishes - covering the whole EEZ region - (Santos et al. 1997), several new records have been published and an updated checklist published (Porteiro et al. 2010). This new dataset covers all confirmed species of bony fish for the Azorean EEZ (Barcelos et al. 2020).

**Data set 1.** 

Column label	Column description
ID	sequential ID
TaxonID	Taxon ID
parentNameUsageID	ID of the parental name usage
scientificName	Scientific Name
parentNameUsage	Parent Name Usage
kingdom	kingdom
phylum	phylum
class	class
order	order
family	family
genus	genus
specific epithet	specific epithet
taxon rank	taxon rank
scientific Name Authorship	Scientific Name Authorship

## Supplementary Material

70ED227C-3C10-52AE-A70D-529E243C57AD 10.3897/BDJ.9.e62812.suppl1 Supplementary material 1 Azores' Exclusive Economic Zone (EEZ) actinopterygians species list - supplementary information Data type occurrence details, taxonomic remarks, IUCN status File: oo_510652.xlsx https://binary.pensoft.net/file/510652 Barcelos, LMDB; Azevedo, JMV; Barreiros, JP
